# Epidemiological and genomic analysis of the dengue virus isolate from Jeddah, Saudi Arabia: Implications for future therapeutic development

**DOI:** 10.1371/journal.pone.0351649

**Published:** 2026-07-16

**Authors:** Shymaa Damfo, Eitezaz Abdulshakoor Zaki, Mohammed Yahya Marouf, Mohammed Abdulmajeed Alghamdi, Khader Saleh Alghamdi, Bader Mohammed Alqarni, Mona A. Almusawi

**Affiliations:** 1 Department of Pharmacognosy and Pharmaceutical Chemistry, College of Pharmacy, Taibah University, Madinah, Kingdom of Saudi Arabia; 2 Health and Life Research Center, Taibah University, Madinah, Saudi Arabia; 3 Senior Specialist, Molecular Biology Department, Jeddah Regional Lab, Jeddah, Saudi Arabia; 4 Lab Technician, Molecular Biology Department, Jeddah Regional Lab, Jeddah, Saudi Arabia; 5 Research Unit, Assistant Agency for Preventive Health, Ministry of Health, Riyadh, Saudi Arabia; Hazara University, PAKISTAN

## Abstract

**Background:**

Dengue fever is one of the major mosquito-borne diseases worldwide, and studies have shown that more than half of the world population could be at risk of dengue in the future. To date, there are no universally effective vaccines or specific antiviral treatments for dengue virus (DENV). Dengue fever has reported in the Western region of Saudi Arabia since 1994. A comprehensive understanding of the molecular epidemiology of the DENV in Saudi Arabia, with implications for therapeutic development, is still absent.

**Aim:**

The present study aimed to integrate analysis of the circulating DENV genome and mutations of the most prevalent dengue serotype in the therapeutic context.

**Methods:**

Retrospective data were used to conduct analysis of dengue infections in Jeddah city in Saudi Arabia from January to October 2024. All Saudis, non-Saudis, and Hajj and Umrah visitors with a confirmed diagnosis of dengue fever were included in the study. The demographic factors and DENV serotypes were explored. The genome of the most prevalent DENV serotype was sequenced using Oxford Nanopore Technology. Closely related strains were searched using the BLAST NCBI tool, and the NGPhylogeny server was used to analyze the phylogenetic relationship. In order to provide a biologically appropriate template for drug-design initiative, the dengue structural protein (prM) of an isolated strain was optimized through receptor modeling and mutation analysis using ICM tool.

**Results:**

Analysis of the circulating DENV serotypes showed that higher incidence rates occurred in young adults, males, and from the Middle Eastern region of origin. The majority (95.3%) of confirmed cases were caused by the serotype DENV-2. A high nucleotide sequence identity was noted for strains originating from Pakistan (99.68%). Five ligands containing nitrogen-rich chemical scaffolds (piperidine, azoles, and sulfonamides) with a variety of substituents were identified by the study as possible binding hits to both wild-type and mutant prM receptor structures. Although of limited sample size and preliminary docking results, the epidemiological and molecular data generated in the study provide useful information for epidemic preparedness and possible therapeutic interventions.

## 1. Introduction

Dengue virus (DENV) belongs to the *Orthoflavivirus* genus of the *Flaviviridae* family. Dengue disease is transmitted by females of the *Aedes* mosquito species*,* most commonly *A. aegypti*. Clinically, dengue infection can range from mild dengue fever to severe plasma leakage with hemorrhagic manifestations. Such conditions may trigger dengue shock syndrome and dengue hemorrhagic fever. According to a modeling study [1], more than half of the world’s population could be at risk of DENV in the future. This estimation was based on several factors, including climate change, global population density, urban growth and extents [2], distribution of the *Aedes* mosquito species [3], and rainfall accumulation [4]. The DENV genome is an ~ 11 kb, single-stranded, positive-sense RNA molecule with 5’ and 3’ untranslated regions (UTRs) flanking a single open reading frame (ORF) that codes for three structural (C, prM/M, and E) and seven non-structural (NS1, NS2A, NS2B, NS3, NS4A, NS4B, and NS5) proteins.

Dengue has been prevalent in Jeddah city in the Westernregion of Saudi Arabia since 1994 [5]. Serious public health concerns have been raised by a recent increase in the incidence of infections caused by DENV, in the Middle East, including Afghanistan [6], Pakistan [7], Oman [8] Sudan [9] and Saudi Arabia, which all reported local DENV transmission. The overall seroprevalence in Saudi Arabia ranges up to 50% in a few districts [10–12]. The major predisposing factors are overcrowded housing, rapid urbanization, poor sanitation, and stagnant water, which create mosquito breeding grounds. Additionally, conditions such as diabetes, hypertension, pregnancy, immunosuppression, and chronic disease conditions significantly increase the chances of secondary infections and mortality [12–17]. Peak occurrences of DENV infections are mostly reported during late spring and early summer. This time of the year is favorable for the mosquito due to increased levels of rainfall and humidity, and high temperatures [14,18,19].

Furthermore, the annual influx of Hajj and Umrah pilgrims from dengue-endemic countries plays a role in the introduction and spread of the disease in Jeddah [19–22], which is a key stop for pilgrims due to its international airport and proximity to Makkah. Since 2022, more than eight million pilgrims have traveled to Saudi Arabia for Umrah [23], and the number of visitors is expected to increase in the future. In 2023, the number of Hajj pilgrims from other countries was 1,660,915 [24]. Most pilgrims (63.5%) come from Asian countries, including Indonesia and India, where DENV infections are endemic [25,26].

Several studies have identified the most prevalent serotype and obtained the whole genome sequences for DENV strains commonly found in Saudi Arabia [27–29]. However, none of these studies have advanced understanding beyond genomic and epidemiological analysis to exploit the data for the design of therapeutics. Epidemiological data provides evidence-based understanding of the patient population and its characteristics in order to measure the range of efficacy and safety during drug development once the product is on the market [30]. The previous literature has collected available genome sequences of 11 different viruses for analysis and to prioritize drugs against SARS‑CoV‑2 [31]. In addition, several recent studies obtained and analyzed patient samples to perform genome sequencing of circulating influenza viruses in order to conduct phylogenetic analysis and examine mutations, followed by structural prediction and docking analysis to examine influenza targets against specific ligands, such as antiviral drugs or siliac acid [32,33]. These papers are examples of how epidemiological and genomic data can be exploited to prioritize ligands by molecular docking, with future implications for the monitoring of outbreaks or therapeutic management.

Earlier research studies have reported the epidemiology and genomic analysis of DENV and used the data to conduct structure-based studies on DENV inhibitors. A study by Racherla et al. (2022) collected ~4,000 samples from dengue patients in South India [34]. The study conducted epidemiology and molecular characterization and identified potential inhibitors of the envelope protein. Four ligands were identified as potential inhibitors, with docking (extra-precision glide, XPG) scores ranging from −9.632 to −8.675 kcal/mol. Another study, conducted in Pakistan, collected 100 positive samples of dengue infections and carried out RNA extraction and serotyping, sequencing, and bioinformatic analysis [35]. In the study, the researchers carried out homology modeling to predict the 3D structure of the envelope protein and estimate the affinity of two ligands (Arbidol and Quercetin) toward the target to give insight into the binding interactions. Another recent study conducted epidemiology and genomics analysis, followed by an assessment of the effects of mutation in binding of the DENV envelope protein to the receptor—which mediates the attachment and entry of the virus into cells—using *in silico* docking analysis [36]. All of the aforementioned studies were conducted in endemic DENV regions, such as India, Pakistan, and Iran, and their findings show the importance of ongoing epidemiology and genomic studies to monitor mutations and their distributions and to understand the implications for therapeutic interventions.

To the best of our knowledge, no previous studies have been conducted in the region of Saudi Arabia. This study was conducted to carry out genomic surveillance and predict the effects of mutations of the most prevalent serotype in order to understand the implications for therapeutic intervention. Thus, the present study aimed to determine the cumulative incidence and distribution of DENV infections over the study period. This study aimed to carry out genome sequencing of the most prevalent serotype and to exploited the use of the isolated sequence to perform phylogenetic analysis, multiple sequence alignment, protein prediction, and *in silico* identification of the ligand targeting the DENV protein. As a case study, this work translated prM protein as a target receptor for further analysis. At the molecular level, DENV maturation involves furin-mediated cleavage of the precursor membrane protein (prM), a 166-amino-acid structural protein, resulting in the formation of mature virions. Recent studies indicate that partially mature prM⁺ virions can contribute to disease pathogenesis during primary infection and that antibodies targeting prM can effectively neutralize DENV infection [37]. This work translated prM protein from the recent sequence isolated in this study from Saudi Arabia to predict the protein structure and conduct subsequent analysis. Moreover, the template protein receptor was mutated based on the isolated sequence and used to evaluate the binding of small-molecule ligands to both wild and mutated prM DENV proteins.

## 2. Materials and methods

### 2.1. Study population and design

This study analyzed retrospective, fully anonymized data obtained from Jeddah regional laboratory, and no direct interaction with participants occurred. All data were deidentified by the Jeddah regional laboratory institution of the Ministry of Health of Saudi Arabia, which receives blood samples from patients with dengue infection from hospitals in Jeddah city. The research team accessed data for research purposes and analysis between 04/24/2024 and 10/31/2024 after obtaining ethical approval. We analyzed laboratory-confirmed dengue cases from 01/01/2024 to 10/31/2024, representing the most recent complete dataset available at the time of the study. Saudis, non-Saudis, and Hajj and Umrah visitors present in the region during the study period were included to calculate the cumulative incidence. Nationality data were used for analysis; however, no classification based on pilgrimage status was used. Suspected cases without lab confirmation and duplicated samples were excluded from the study. The demographic factors section included age, gender, nationality, region of origin, previous history of dengue fever, and the date of disease diagnosis.

### 2.2. Ethical approval

Ethical approval was obtained from the Ethics Committee of the Ministry of Health in Saudi Arabia (Number 24-42E). The Ethics Committee of the Ministry of Health in Saudi Arabia provides national-level ethical approval. The data were fully anonymized, and no direct interaction with participants occurred. All data were deidentified before any further analysis was carried out.

### 2.3. Case definition

All of the cases included in the analysis were laboratory-confirmed dengue cases, defined according to the World Health Organization (WHO) dengue guidelines. Dengue is classified as either dengue without warning signs, dengue with warning signs, or severe dengue, based on clinical presentation and laboratory confirmation (WHO, 2009).

### 2.4. Statistical analysis

Data analysis was performed using the Jamovi software (version 2.3). Descriptive statistics were used to summarize categorical variables as frequencies and percentages. To calculate cumulative incidence, as a denominator we used the projected 2024 residential population of Jeddah (4,081,307), derived from the 2022 census figure and applying the annual regional growth rate of 4.3%, and expressed the incidence per 100,000 population. Cumulative incidence was used to estimate population risk over the study period because the data were obtained retrospectively from the surveillance system and the individual follow-up times were not available to calculate person-time incidence rates. The associations between the demographic variables and DENV serotypes were analyzed using Fisher’s exact test. Odds ratios (ORs) with 95% confidence intervals were calculated, with statistical significance set at a value of < 0.05.

### 2.5. Virus strains and sequencing

DENV RNA was extracted from patient serum using an ExiPrep™ 96Viral DNA/RNA Kit (Bioneer, Korea), following the manufacturer’s instructions. For each sample, 200 µL of serum was loaded into the well of the extraction system. Nucleic acids were bound to magnetic beads after lysis and, subsequently, a washing step was performed. The purified RNA was eluted and subjected to one-step real-time RT-PCR assay using a VIASURE Dengue Serotyping Real Time PCR Detection Kit (CerTest Biotec, Spain), which detects amplified DENV serotypes (DENV-1 to DENV-4). The assay uses specific primers and a fluorescence probe that targets the high conserved regions of the NS5 gene (DENV-1), envelope gene (DENV-2), prM gene (DENV-3), and NS2A gene (DENV-4).

A total of 15 µL of the reaction mixture (supplied with the kit) was mixed with the rehydration buffer. This step was followed by adding 5 µL of purified nucleic acid to each well of the reaction. Positive control (non-infectious synthetic lyophilized cDNA) and negative control (non-template control) supplied with the kit were included to ensure the quality and validity of the results. Thermal cycling and fluorescence detection were performed using an RT-PCR LightCycler® 480 II system (Roche, Germany), set up for thermal cycling by the following steps: A) reverse transcription step (one cycle for 15 min at 45ºC), B) initial denaturation step (one cycle for 2 min at 95ºC), and C) denaturation (45 cycles for 10 sec at 95ºC) and annealing/extension (50 sec at 60ºC). The fluorescence signal was collected during the extension step through the FAM (DENV 1 and 3); ROX (DENV 2 and 4); and HEX, JOE, or VIC channels (internal control [IC]). The PCR products were subjected to cycle sequencing using a Bigdye Terminator V3.1 Reaction Cycle Kit on an ABI 3500 Automatic Sequencer (Applied Biosystems, USA).

### 2.6. Genome sequence of the most prevalent serotype

Viral RNA of the most common serotype was extracted in a previous step and amplified by PCR for library preparation using an Oxford Nanopore platform. Sequencing was performed on a GridION (Oxford Nanopore Technologies, UK), following an amplicon strategy targeting viral RNA. RNA samples exhibiting Ct values below 20 were diluted between 10- and 100-fold before reverse transcription to avoid reaction inhibition and to ensure optimal efficiency. cDNA synthesis was carried out using SuperScript™ IV VILO™ Master Mix (Invitrogen; Cat. No. 11756500), in accordance with the manufacturer’s instructions.

#### 2.6.1. Multiplex PCR.

The primer panel selected for each serotype was divided into two separate primer pools. Accordingly, two PCR reactions per sample were prepared, one containing primer pool 1 and the other containing primer pool 2. Primers from each pool were diluted to a working concentration of 100 μM and thoroughly mixed as described above. Multiplex PCR amplification was performed using Q5® Hot Start High-Fidelity DNA Polymerase (New England Biolabs; Cat. No. M0493L). For each primer pool, amplicons were generated in a 50-μL reaction containing 5 μL of cDNA template, with each primer included at a final concentration of 0.015 μM. The thermal cycling conditions were as follows: initial denaturation at 98°C for 30 s followed by 30 or 35 cycles—depending on the sample Ct value (30 cycles recommended for Ct < 30 and 35 cycles for Ct ≥ 30)—consisting of denaturation at 98°C for 10 s, annealing at 50°C for 15 s, and extension at 72°C for 5 min, with a final extension at 72°C for 10 min. PCR products from pools 1 and 2 were verified using an Agilent bioanalyzer and subsequently pooled in equimolar amounts per sample.

#### 2.6.2. Library preparation.

Sequencing libraries were prepared using a Ligation Sequencing Kit (SQK-LSK114; Oxford Nanopore Technologies) according to the manufacturer’s instructions for amplicon sequencing. Briefly, DNA repair and end-prep were performed using the NEBNext® Ultra™ II End Repair/dA-Tailing Module. End-prepped DNA was purified using AMPure XP beads, and the sequencing adapters were ligated using NEBNext® Quick T4 DNA Ligase. The adapter-ligated libraries were cleaned using AMPure XP beads and eluted in Elution Buffer (EB) supplied with the kit. The final library concentration was measured using Qubit, and the loading quantity was adjusted according to the Oxford Nanopore recommendations for R14.4.1 flow cells.

#### 2.6.3. Nanopore sequencing (GridION, R14.4.1 Flow Cell).

Libraries were loaded onto R14.4.1 (V14 chemistry) flow cells and sequenced on a GridION Mk1 device (Oxford Nanopore Technologies). Sequencing runs were configured using MinKNOW using the standard high-accuracy settings. Real-time monitoring was performed to assess pore occupancy and yield and to read quality.

#### 2.6.4. Basecalling and read processing.

The raw POD5 files were basecalled using Dorado (latest stable release; Oxford Nanopore Technologies) in super-accuracy mode. Reads were filtered with a minimum Phred quality score threshold of Q10.

#### 2.6.5. Analysis using genome detective.

Basecalled and quality-filtered Oxford Nanopore reads generated from serotype-specific multiplex PCR amplicons were analyzed using Genome Detective for genome assembly and genotyping. Processed FASTQ files (minimum Q score ≥ 10) were uploaded to the Genome Detective platform, where reads were automatically filtered, reference-mapped, assembled, and polished to generate consensus genomes. Serotype classification and genotyping were assigned based on curated DENV reference databases integrated within the platform. Assembly quality metrics, including genome coverage, depth, and consensus completeness, were reviewed prior to downstream phylogenetic and variant analyses.

### 2.7. Sequence and phylogenetic analysis

The assembled genome was deposited in GenBank NCBI SRA under accession number SRR35750115. The genome of the isolated sequence serotype (DENV-2-Jeddah-2024) was initially analyzed for similarity using the BLAST tool (http://www.ncbi.nlm.nih.gov/BLAST/) [38] with the default search settings. Closely related DENV-2 sequences with high similarity to the isolated one were selected for additional analysis. We selected 25 reference strains based on the BLAST similarity thresholds (highest percent identity) to our query sequence to ensure focused analysis and inclusion of the most relevant isolates. The BLASTX tool (part of the BLAST suite developed by NCBI) [38] was used to translate the nucleotide sequence of the DENV-2-Jeddah-2024 isolate into a protein sequence. The Nucleotide and Protein BLAST tools were used to align the query sequence with closely related sequences in order to determine the identity percentage. Multiple sequence alignment (MSA) of nucleotide sequences was performed using Clustal Omega [39].

In addition to aligning the obtained sequence with the global sequences in NCBI, we retrieved sequences circulating in the region to compare them with the identified sequence. The DENV genomes available in the NCBI Virus database were filtered based on their geographic location (Saudi Arabia) and downloaded for further analysis. The sequence of the DENV-2-Jeddah-2024 isolate was aligned with sequences isolated locally to capture genetic variations with closely related sequences circulating in the region.

Phylogenetic analysis was conducted and distance calculations were determined using the NGPhylogeny service [40]. This list of 25 DNA sequences of reference strains was submitted to NGPhylogeny.fr as a FASTA file using the PHYML/OneClick tool in the advanced workflow. The workflow was implemented and integrated with MAFFT (version 7.467) for MSA, and alignment trimming was performed with BMGE using the default parameters. The phylogenetic reconstruction was performed using the maximum-likelihood method implemented in PhyML (version 3.3.20190909) under the GTR nucleotide substitution model with gamma-distributed rate heterogeneity (four categories, α estimated) and an estimated proportion of invariant sites. The resulting tree was visualized as an unrooted tree. The phylogenetic robustness was inferred via a bootstrapping procedure with 1,000 replications.

### 2.8. MSA of prM Protein of the Isolated Strain with Reference Strain and Template-Guided Protein Prediction using AlphaFold2

MSA was performed using DENV-2-Jeddah-2024 with the closely related sequence isolated from Saudi Arabia (2019-OK048579.1-Saudi Arabia) for the prediction of mutation in the target protein. This reference strain was identified using the BLAST tool [38] as the closest match to our isolate. The reference sequence was partially available; thus, alignment and comparative analysis were restricted to the available genome, including the genome encoding the prM protein. To determine the three-dimensional (3D) structure of the prM protein of the circulating strain, the AlphaFold2 package was used [41–43]. The amino acid sequence of prM of the isolated strain (DENV-2-Jeddah-2024) was submitted to AlphaFold2 [41–43] using the default settings to predict the prM structure and conformational ensemble. Validation of the predicted structure and alignment of the two structures were carried out using ICM software [44] to compare the AlphaFold2 predicted model and the experimentally solved structure of the prM protein available in the Protein Data Bank (PDB) (PDB: 3C5X). This protein structure (3C5X) was used as the template for AlphaFold2 prediction because it represents the highest-resolution structure of prM currently available in the PDB. Validation parameter of root-mean square deviation (RMSD) was calculated using three different approaches—i) all-atoms, ii) solely carbon atoms, and iii) backbone (N, C, C, and O atoms)—available in the ICM software [44].

### 2.9. prM protein mutations with implications for future therapeutic development

The electron microscopy–derived 3D structure of the DENV-2 prM protein was downloaded from PDB (PDB: 8FE4) to use as a docking template and to predict the structural impact of each mutation that exists in DENV-2-Jeddah-2024 on drug-target interaction. The structure 8FE4 was selected as the docking template due to the presence of the bound control ligand, allowing docking validation. Mutated residues were identified by MSA using Clustal Omega [39]. The structure was optimized using ICM [44] by protonation, fixing backbone and sidechain atom names, optimizing His/Pro/Asn/Gln/Cys, assigning secondary structure, and energy minimization.

To perform molecular docking, the receptor grid was defined based on the druggable binding site of the original ligand found in the earlier work [37]. The control structure (prM13) [37] was docked against the wild type (PDB: 8FE4) and the mutant structure DENV-2-Jeddah-2024 using the fast Fourier transform method for protein–protein docking [45]. Following this, chemical molecules (fragments) of the Diamond-SGC poised library [46] were utilized for assessing the binding of the ligands to the active site of the original structure. The grid box size was chosen to be approximately 60 × 60 × 60 Å to include all ligand conformations, and the grid center was determined as the mean of the X, Y, and Z coordinates (89, 94, 46). Docking was performed using ICM Molsoft with default parameters of docking run and scoring threshold (−32). Default settings of ligand preparation protocol were kept, where partial charges were automatically allocated, hydrogen atoms were added, and 2D structures were transformed into optimal 3D conformations. The compounds were clustered based on their docking scores, and the set of compounds (60) including favorable binding energy was further docked against the mutant structure to evaluate the difference in binding energy. Compared to purely empirical scoring methods, the ICM score uses a physics-based energy function obtained from a GBSA/MM model enhanced with a directed hydrogen bonding term, enabling computational estimation of relative binding energies.

A docking score ≤ ˗32 was determined as the cut-off based on the ICM docking protocol [47], as this value or lower generally indicates favorable binding. As the docking score threshold may vary depending on the target, this cut-off was applied as a relative selection criterion to prioritize potential binders rather than as an experimentally validated cut-off specific to prM.

## 3. Results

### 3.1. Demographic characteristics of the population affected by dengue

A total of 192 dengue cases reported to Jeddah regional laboratory, Saudi Arabia, in the period January to October 2024 were included in the analysis. The demographic data and dengue fever factors of cases are illustrated in Table 1. The majority of cases were observed among adults aged 18–39 years (62.0%), whereas the lowest proportion was recorded in children < 18 years (1.0%). Males constituted 89.1% of the cases, with non-Saudis representing the majority (74%). In terms of region of origin, approximately half of the cases occurred among Middle Eastern individuals (47.4%), followed by South Asian individuals (41.1%). With respect to patients with a history of dengue fever, 118 (61.5%) cases provided this information. Of these, 43.2% reported a previous history of the disease, as shown in Table 1. The results indicate that during the study period, dengue infection mostly occurred in young non-Saudi males from the Middle East and South Asia. All of the dengue cases analyzed in the study are listed in S1 Table.

**Table 1:** Demographic and dengue fever factors among cases.

Variable	N (192)	%
**Age Group:**		
< 18	2	1.0
18-39	119	62.0
40-59	57	29.7
≥ 60	14	7.3
**Gender:**		
Male	171	89.1
Female	21	10.9
**Nationality:**		
Saudi	50	26.0
Non-Saudi	142	74.0
**Region of origin:**		
Middle East	91	47.4
Africa	6	3.1
South Asia	79	41.1
Southeast Asia	2	1.0
South American	1	0.5
Unknown	13	6.8
**History of Dengue Fever:**		
Yes	51	26.6
No	67	34.9
Unknown	74	38.5

### 3.2. Cumulative Incidence of DENV Serotypes

**Fig 1:**
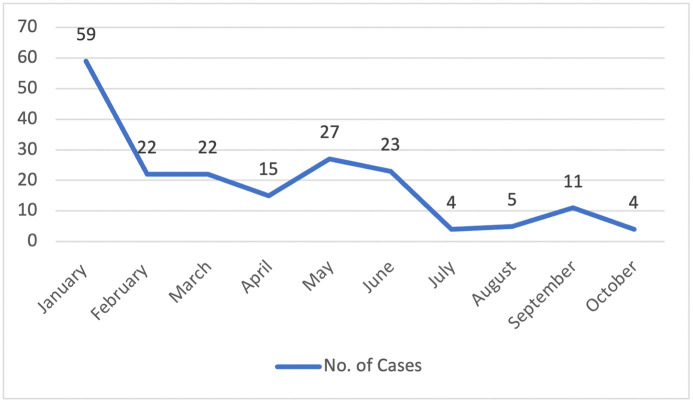
The monthly distribution of dengue cases.

The distributions and cumulative incidences of DENV serotypes are presented in Table 2. Most reported cases (95.3%) were caused by DENV serotype-2 (DENV-2), which corresponded to a cumulative incidence (CI) of 4.48 per 100,000. A small proportion of cases were attributed to no prior history and DENV-3, which accounted for 1.0% and 3.6%, respectively, with very low CI (0.05 and 0.17 per 100,000). No cases of DENV-4 were identified in this study. The overall CI of dengue in Jeddah during the study period was 4.70 per 100,000 population. The number of dengue cases in each month during the study period is illustrated in Fig 1. The highest number of cases was observed in January (59 cases). Another subsequent increase was observed in May (27 cases) and June (23 cases), followed by a sharp decrease to the lowest level of the study period in July (four cases).

**Table 2:** Distribution of dengue fever serotypes among confirmed cases.

DENV Serotype	No.	%	Cumulative Incidence per 100,000^a^
DENV-1	2	1.0	0.05
DENV-2	183	95.3	4.48
DENV-3	7	3.6	0.17
DENV-4	0	0	0.00
Total	192	100	4.70

^a^Cumulative Incidence calculated using projected 2024 Jeddah population (4,081,307), based on the 2022 census figure from citypopulation.de and applying a 4.3% annual regional growth rate (GASTAT), expressed per 100,000.

### 3.3 Relationships between demographic factors and DENV serotypes

The relationships between the demographic factors and DENV serotypes are summarized in Table 3. There was a statistically significant relationship between region of origin and DENV serotype (p = 0.012), indicating significant variation across regions. The majority of reported DENV-2 cases were among Middle Eastern and South Asian patients, compared with the other serotypes, which were few and scattered among the regions. Additionally, a borderline significant relationship was found between nationality and DENV serotype (p = 0.053). Non-Saudi cases had approximately four times higher odds of DENV-2 than Saudis (OR = 3.83, 95% CI 0.987–14.9). No statistically significant relationships were found between age group or gender with dengue serotype (p = 0.874 and p = 0.256, respectively). The prevalence of DENV-2 among males was more than two times higher than among females, but the relationship was not statistically significant (OR = 2.47, 95% CI 0.478–12.7).

**Table 3:** The relationships between demographic factors and the DENV serotype.

Factor	DV Serotype N(192)		Fisher’s exact test		95% CI
**DENV-2**	**Other Serotypes^a^**	p value	OR	
**Age Group:**			0.874	–	–
< 18	2	0			
18-39	112	7			
40-59	55	2			
≥ 60	14	0			
**Gender:**			0.256	2.47	0.478–12.7
Male	164	7			
Female	19	2			
**Nationality:**			0.053	3.83	0.987–14.9
Saudi	45	5			
Non-Saudi	138	4			
**Region of origin:**			0.012	–	–
Middle East	83	8			
Africa	6	0			
South Asia	79	0			
Southeast Asia	1	1			
South American	1	0			
Unknown	13	0			

^a^Other Serotypes include DENV-1 and DENV-3.

### 3.4. Genome sequence and phylogenetic analysis of the most prevalent serotype

**Fig 2:**
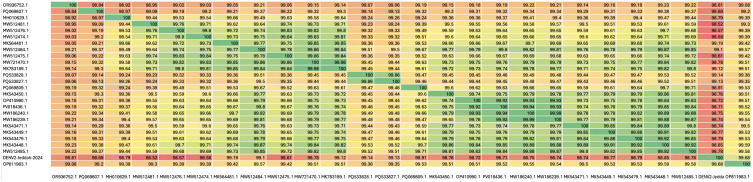
Sequence identity heatmap illustrating pairwise percent identity in nucleotide between the DENV-2-Jeddah-2024 strain and closely related global sequences in NCBI. MSA and pairwise percent identity were calculated using Clustal Omega, and a heatmap was generated using Excel software.

**Fig 3:**
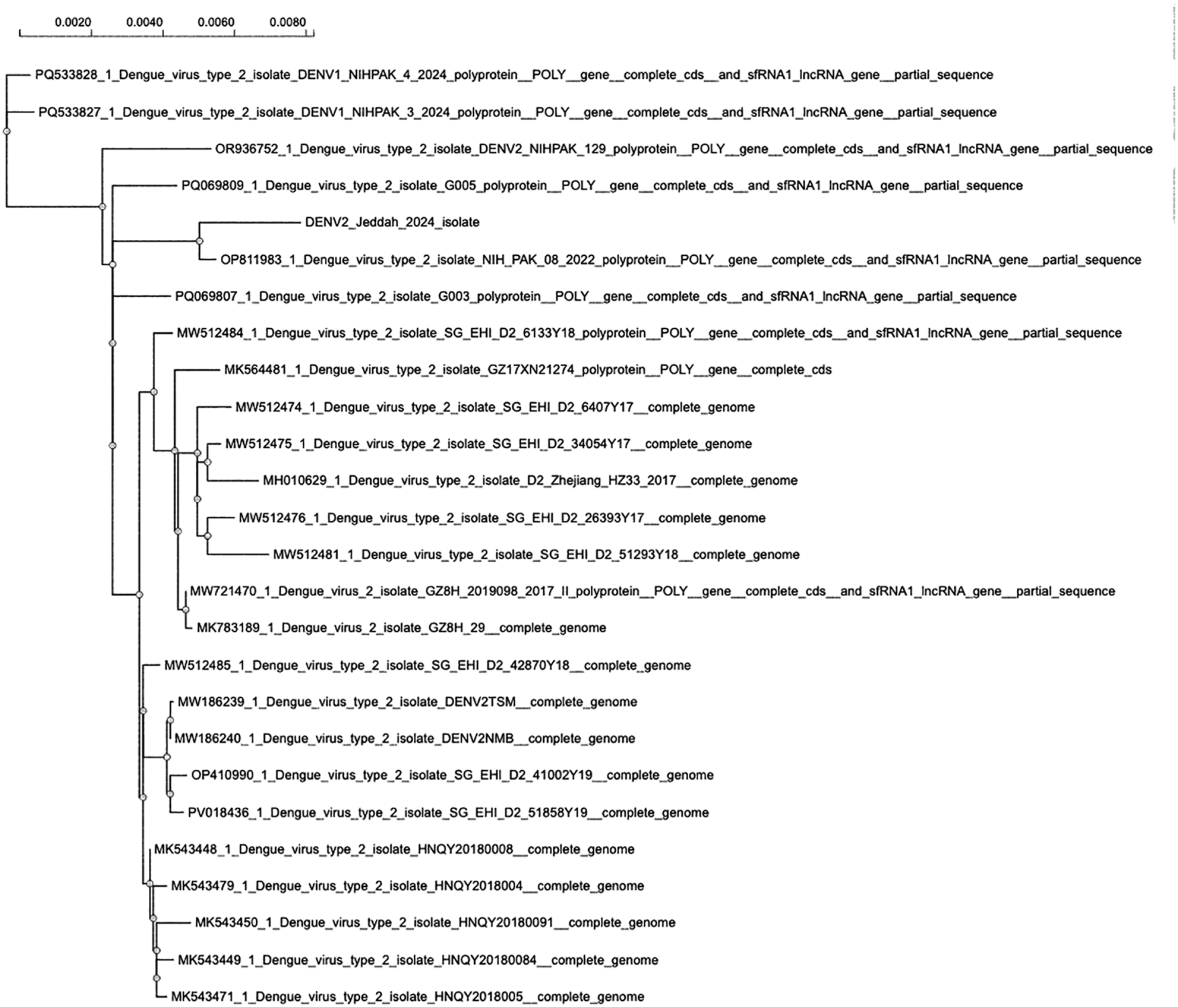
Phylogenetic tree of DENV-2-Jeddah-2024 strain. The tree was created using the NGPhylogeny service [40]. Closely related sequences were retrieved using the BLAST tool. All sequences are labelled with their GenBank accession numbers. The analysis performed using the PHYML/OneClick tool within an advanced workflow, integrated with MAFFT for alignment. Trimming was done with BMGE using default settings. The maximum-likelihood method in PhyML was applied for phylogenetic reconstruction, utilizing the GTR nucleotide substitution model with gamma-distributed rate heterogeneity and an estimated proportion of invariant sites. The tree is unrooted Phylogenetic robustness was assessed through a bootstrapping procedure with 1000 replicates.

The genome of the most prevalent serotype (DENV-2) was sequenced. The assembly of all sequenced overlapping segments derived from RT-PCR yielded a genome comprising 10,664 nucleotides. The identity matrix of nucleotides and amino acids of the DENV-2-Jeddah-2024 isolate was then compared to highly similar isolates based on their identity percentages (25 DENV-2 strains are presented in Table 4) available in GenBank. All closely similar isolates to our isolated sequence resulting from the BLAST search are listed in S2 Table. The comparative genomic analysis showed a greater than 99% sequence identity with the strains reported in Table 4. Recovery of terminal regions (5′ and 3′ UTRs) may be incomplete due to limitations inherent to the sequencing and assembly approach. However, the isolate showed high nucleotide and protein sequence similarity to previously reported DENV-2 as shown in Table 4. The high sequence identity was particularly notable for strains originating from Pakistan, Singapore, and China. The highest nucleotide identity was observed in strain 2022-OP811983.1-Pakistan (99.68%), followed by 2021-PQ069807.1-Pakistan (99.42%), 2018-MK543448.1-China (99.37%), and 2018-MW512485.1-Singapore (99.36%). Pairwise comparisons amongst each sequence were illustrated as a heatmap, as shown in the Fig 2. The phylogenetic tree of the DENV-2-Jeddah-2024 strain is presented in Fig 3. Amino acid identities for the top sequences in Table 4 ranged from 99.3% to 99.9%, showing a high level of protein conservation. Globally, the lowest nucleotide identity (99.10%) observed among these strains was with strain 2017-MH010629.1-China, while the lowest protein identity (99.3%) was recorded with 2023- OR936752.1- Pakistan strain.

**Table 4:** Nucleotide and amino acid percent identity of DENV-2-Jeddah-2024 isolate genome compared to closely related strains. (A) Isolate compared to global references (B) Isolate compared to previously reported sequences in Saudi Arabia.

Strain	Nucleotide Per. Ident (%)	Amino Acid Per. Ident (%)
(A) compared to closely related international strains		
2022- OP811983.1- Pakistan	99.68	99.85
2021- PQ069807.1- Pakistan	99.42	99.38
2018- MK543448.1- China	99.37	99.79
2018- MW512485.1- Singapore	99.36	99.79
2018- MW512484.1- Singapore	99.35	99.76
2021- PQ069809.1- Pakistan	99.33	99.79
2019- MW186240.1- Singapore	99.33	99.76
2019- MW186239.1- Singapore	99.32	99.76
2019- OP410990.1- Singapore	99.32	99.79
2018- MK543479.1- China	99.31	99.65
2019- PV018436.1- Singapore	99.31	99.76
2017- MW721470.1- China	99.3	99.79
2018- MK543450.1- China	99.3	99.59
2018- MK543449.1- China	99.29	99.71
2017- MK783189.1- China	99.28	99.79
2018- MK543471.1- China	99.26	99.59
2023- OR936752.1- Pakistan	99.24	99.26
2017- MW512475.1- Singapore	99.20	99.73
2017- MK564481.1- China	99.19	99.65
2017- MW512474.1- Singapore	99.17	99.73
2017- MW512476.1- Singapore	99.16	99.76
2024- PQ533828.1- Pakistan	99.14	99.68
2024- PQ533827.1- Pakistan	99.13	99.73
2018- MW512481.1- Singapore	99.11	99.73
2017- MH010629.1 China	99.10	99.71
(B) Compared to closely related strains isolated in Saudi Arabia		
2019-OK048579.1-Saudi Arabia	98.00	99.33
1994-AM748169.1-Saudi Arabia	95.83	98.75
1994-AM748163.1-Saudi Arabia	95.42	98.75
1994-AM748174.1-Saudi Arabia	95.00	98.75
1994-AM748173.1-Saudi Arabia	95.00	98.75
1994-AM748172.1-Saudi Arabia	95.00	98.75
1994-AM748171.1-Saudi Arabia	95.00	98.75
1994-AM748170.1-Saudi Arabia	95.00	98.75
1994-AM748168.1-Saudi Arabia	95.00	98.75
1994-AM748167.1-Saudi Arabia	95.00	98.75
1994-AM748166.1-Saudi Arabia	95.00	98.75
1994-AM748165.1-Saudi Arabia	95.00	98.75
1994-AM748164.1- Saudi Arabia	95.00	98.75
1994-AM748160.1-Saudi Arabia	95.00	98.75
1994-AM748158.1-Saudi Arabia	95.00	98.75
1994-AM748156.1-Saudi Arabia	95.00	98.75
1994-AM748155.1-Saudi Arabia	95.00	98.75
1994-AM748153.1-Saudi Arabia	95.00	98.75
2014-KU886707.1-Saudi Arabia	95.00	98.51
2014-KU886706.1-Saudi Arabia	95.00	98.51
2014-KU886705.1-Saudi Arabia	94.81	98.59
2015-KU886713.1-Saudi Arabia	94.81	97.40
2015-KU886712.1-Saudi Arabia	94.81	97.40
2014-KU886709.1-Saudi Arabia	94.81	97.40
2014-KU886708.1-Saudi Arabia	94.81	97.40

### 3.5. MSA of prM Protein of the recent circulating strain and protein structure prediction

**Fig 4:**
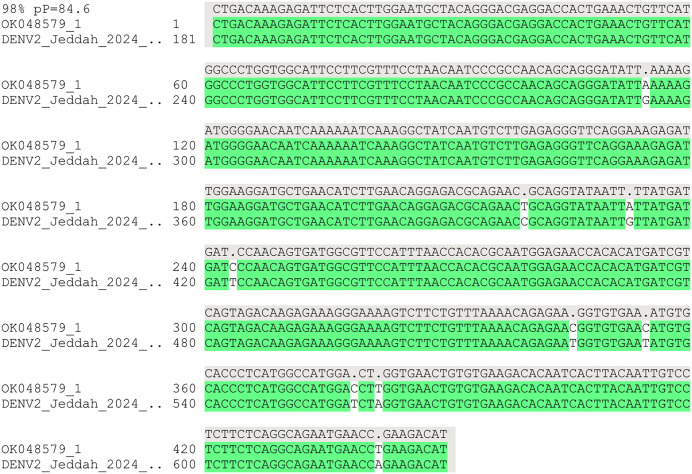
MSA of nucleotide sequences of the newly discovered strain DENV-2-Jeddah-2024 and the previous closely related sequence isolated from Saudi Arabia (2019-OK048579.1-Saudi Arabia). The alignment was based on the available reference genome (~600 nt) identified by the BLAST tool. The figure shows conserved nucleotides denoted by (*). The figure was generated using ICM software [44]. The numbering of DENV-2-Jeddah-2024 was based on the reference sequence [37].

**Fig 5:**
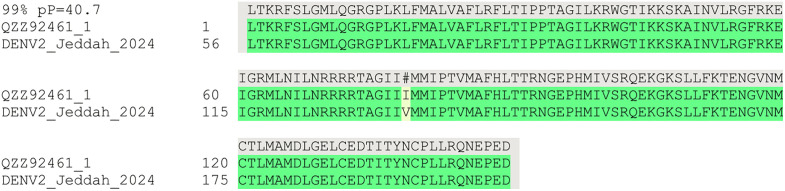
MSA of protein Sequences of the newly discovered Strain DENV-2-Jeddah-2024 and the previous closely related sequence isolated from Saudi Arabia QZZ92461.1 (corresponding to 2019-OK048579.1-Saudi Arabia). The figure shows conserved and variable amino acids, where identical ones are denoted by (*) and variable amino acids are denoted by (:). The MSA was generated using ICM software [44]. The numbering of DENV-2-Jeddah-2024 was based on the reference sequence [66].

The results showed 98% nucleotide sequence identity between our isolate and the 2019 Saudi Arabian strain (OK048579.1), which encodes the structural protein prM (membrane glycoprotein precursor) of DENV-2. The closely related strain was isolated from the Jazan region of Saudi Arabia. The MSA of DENV-2-Jeddah-2024 with this closely related sequence (2019-OK048579.1-Saudi Arabia) showed nucleotide and amino acid conservation, as presented in Fig 4 and Fig 5. There were nine nucleotide mutations when compared to the closely related strain 2019-OK048579.1 from Saudi Arabia. The protein sequence alignment revealed a single substitution at position 137 from Valine to Isoleucine. BLAST analysis showed that residue 137 is highly conserved across available sequences (S3 File), with only two sequences (WJE97932.1 and AJD81325.1) exhibiting the same substitution. However, no evidence was found that this position V137I lies within a known functionally important or structurally constrained region. The functional impact therefore remains unclear and needs further investigations.

**Fig 6:**
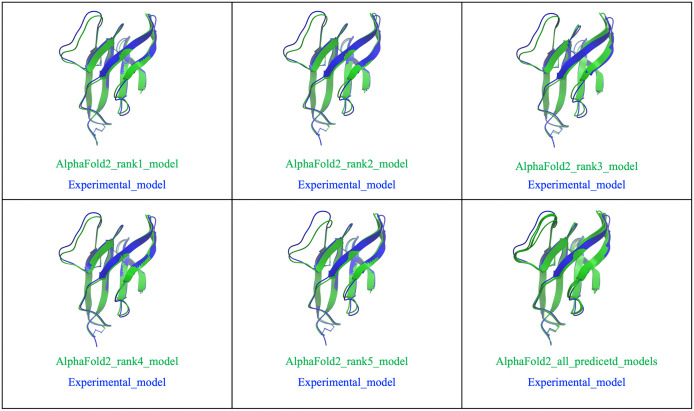
AlphaFold2 prM structures our DENV-2-Jeddah-2024 isolate. Five predicted models of the isolated prM (green) compared with the experimental prM target (PDB 3C5X, blue). Superimposition of structures was performed using ICM software [44].

The 3D structure of the prM isolated sequence was successfully predicted using AlphaFold2 and was assessed based on structural confidence, uniqueness, and internal consistency. Ten models were generated with generally high overall quality (Table 5) but with variations observed mainly in mean pLDDT, max PAE, and uncertainty percentages. The top-ranked conformation showed a mean pLDDT score of 81.36, indicating a high overall prediction confidence. Validation by calculating the RMSD and comparing it with the original structure resulted in an RMSD range from 0.63 to 0.92 Å using alignment of C𝛼 Carbons. Table 6 shows the RMSD with different superimposed parameters, indicating that variation could be as high as 1.68 Å. While model rank 1 was found to be the best in the AlphaFold2 analysis report, model rank 4 performed the best in terms of RMSD value, which necessitates cautious interpretation of the results. We observed positional divergence in the flexible loops of all predicted structures compared to the reference structure, as shown in Fig 6. The variation between AI-predicted models and experimental structure reflects the existence of unusual conformations and the complexity of protein folding, which may contribute to the present constraints in protein structure prediction.

**Table 5:** Summary of AlphaFold2 predicted protein models for prM protein.

Model Rank	Mean pLDDT	Max PAE	pTM	ipTM	pDockQ	Uncertainty	Overall Quality
1	81.36	31.38	0.62	N/A	N/A	3.61%	High
2	79.91	30.94	0.63	N/A	N/A	4.97%	High
3	79.54	31.25	0.62	N/A	N/A	6.91%	High
4	78.75	31.31	0.62	N/A	N/A	4.95%	High
5	78.27	31.19	0.62	N/A	N/A	7.5%	High

**Table 6:** RMSD values (Å) of each predicated model. Each value is a measure of the average distance between the indicated atoms of two superimposed structures (AlphaFold2 predicted model and experimental model). Superimposition was performed by ICM [44] with the following parameters (align residues, visible atoms, and weight iterative superposition).

	RMSD values (Å)				
	Rank_1	Rank_2	Rank_3	Rank_4	Rank_5
**All atoms**	1.54	1.48	1.68	1.43	1.63
**only C*α*** **carbons**	0.76	0.72	0.92	0.63	0.91
**Backbone (N, C*α*, C and O atoms).**	0.75	0.71	0.90	0.62	0.89
**Heavy atoms**	1.33	1.24	1.44	1.17	1.38

### 3.6. prM Protein mutations with implications for future therapeutic development

**Fig 7:**
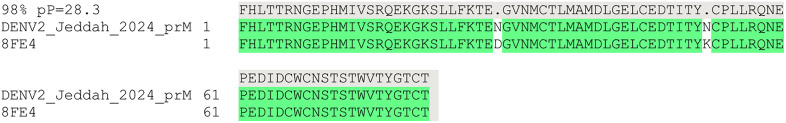
Receptor sequences from the reference and isolated viral strain are aligned using ICM software [43]. The amino acid sequences of the receptor from the original (wild-type) strain (PDB ID 8FE4) and the mutant strain (DENV-2-Jeddah-2024) were aligned. Non-conserved residues are left in colon. The numbering of DENV-2-Jeddah-2024 was based on the reference sequence [37].

**Fig 8:**
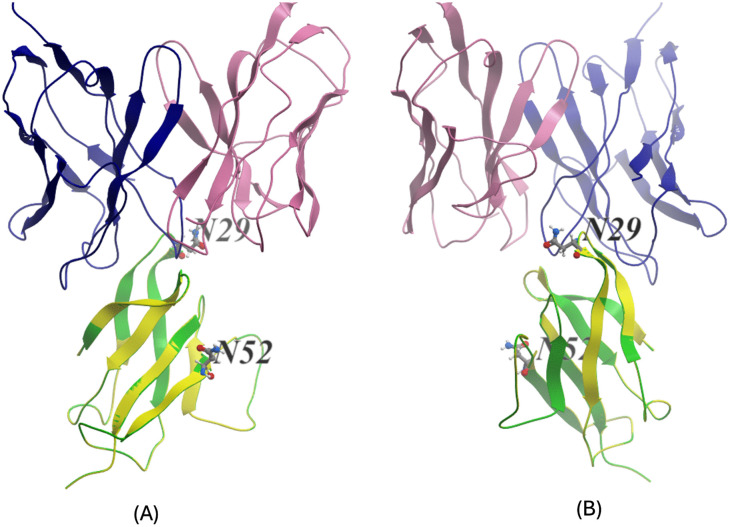
The figure visualizes locations of mutant residues on the DENV prM receptor. (A) back view (B) front view of DENV prM receptor. The prM structure DENV-2-Jeddah-2024 (yellow) is superimposed with (PDB: 8FE4) (green), where the latter is bound to the light chain prM13 antibody (pink), and the heavy chain prM13 antibody (blue).

The experimentally solved electron microscopy–derived 3D structure of the DENV-2 prM protein was obtained from the PDB database (PDB: 8FE4). The protein sequence of the prM protein (PDB: 8FE4) was aligned with DENV-2-Jeddah-2024 to identify amino acid variations. MSA showed two mutant residues: D29N and K52N (Fig 7). In order to visualize locations of these residues on the DENV prM receptor, mutations were mapped onto 3D structure (PDB: 8FE4) using ICM software [44]. As shown in Fig 8, the D29N mutation lies at the protein-antibody binding interface, whereas K52N was at an exposed and spatially distant site from the binding site. *In silico* mutagenesis showed a slight difference in dGbind (ΔG) when we quantified the effect of mutations on binding affinity (Table 7). The ddGbind (ΔΔG) values range from −0.07 to 0.05 kcal/mol.

**Table 7:** Effect of mutations on binding affinity. The difference in Gibbs free energy between the wild and mutant protein upon ligand binding. Positive values indicate destabilisation, while zero or negative values indicate stabilisation. The values were calculated using ICM software.

Ligand	residue	wt	mut	ddGbind	dGbind wt	dGbind mut	Lig repuls^a^	SC repuls^b^
57	29	asp	asp	0.05	−12.63	−12.63	0.00	0.05
	52	lys	asp	0.03	−12.63	−12.60	0.00	0.03
4	29	asp	asp	0.05	−13.65	−13.65	0.00	0.05
	52	lys	asp	−0.01	−13.65	−13.66	0.00	0.03
53	29	asp	asp	0.02	−21.31	−21.31	0.00	0.05
	52	lys	asp	0.03	−21.31	−21.28	0.00	0.03
43	29	asp	asp	0.05	−10.95	−10.95	0.00	0.05
	52	lys	asp	−0.07	−10.95	−11.03	0.00	0.03
24	29	asp	asp	0.05	−14.35	−14.35	0.00	0.05
	52	lys	asp	0.00	−14.35	−14.35	0.00	0.01

a Lig repuls = protein–ligand clashes

b SC repuls = protein side-chain clashes

**Fig 9:**
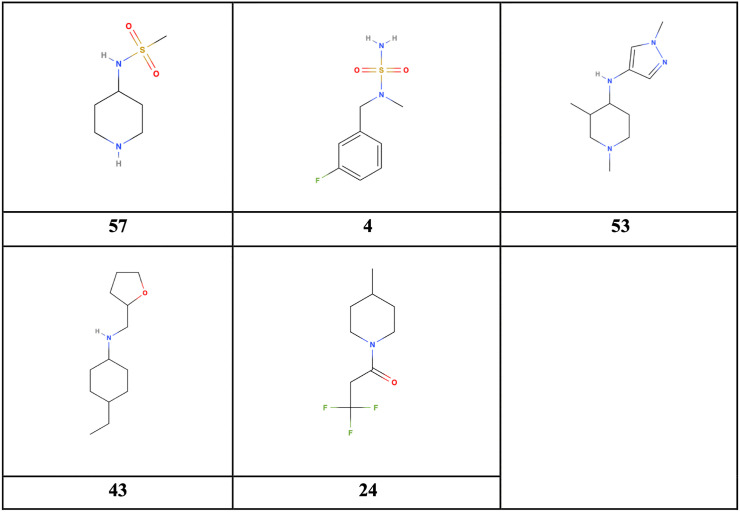
The two-dimensional structures of the chemical fragments that were determined to be the top hits in the virtual screening campaign. These five top chemical ligand structures commonly bind to both prM proteins (origin and mutant receptor structures).

To perform docking analysis, the same procedure, which included side-chain optimization, energy minimization, and protonation state assignment, was used to prepare the origin (wild-type; 8FE4) and mutant (DENV-2-Jeddah-2024) receptor structures. Docking of the control structure (prM13) against both targets showed similar ICM docking scores (−22.19, 8FE4) (−22.11, DENV-2-Jeddah-2024). We found eight chemical ligands that showed docking scores better than the control (Table 8). Moreover, docking analysis revealed that the D29N and K52N mutations resulted in generally predicted lower affinities between chemical ligands and the target. Based on the binding energy cut-off of ˗32, eight ligands were predicted as promising candidates for the origin prM protein (8FE4), while only five ligands had favorable docking scores to the mutant prM protein (DENV-2-Jeddah-2024), as shown in Table 8. Interestingly, the five ligands, which are a series of nitrogen-rich scaffolds (piperidine, azoles, and sulfonamides) with diverse substituents, as presented in Fig 9, were classified based on their ICM scores as top-ranked docking hits to both receptor structures (wild-type; 8FE4) and the mutant structure (DENV-2-Jeddah-2024). Interaction analysis showed that chemical ligands were bound to prM mainly by hydrophobic, van der Waals, and π–cation interactions (S4 Fig).

**Table 8:** Docking results scores against both wild-type (8FE4) and mutant prM receptor (DENV-2-Jeddah-2024) structures after screening of molecules. Small molecules are ranked according to their ICM score against both receptors, where a stronger predicted binding affinity is indicated by more negative ratings.

Rank	Chemical Ligand	ICM Docking score prM DENV-2 target (8FE4)	Chemical Ligand	ICM Docking score prM target (DENV-2-Jeddah-2024)
**1**	57	−45.36	57	−46.08
**2**	4	−37.95	4	−36.60
**3**	53	−35.92	53	−33.85
**4**	43	−34.14	43	−32.78
**5**	2	−32.87	24	−32.74
**6**	7	−32.83	Control prM13	−22.11
**7**	24	−32.44		
**8**	14	−32.07		
**9**	Control prM13	−22.19		

## 4. Discussion

In Saudi Arabia, dengue cases and outbreaks are mostly reported in the southwestern (Jazan) and western (Jeddah) regions. Despite frequent outbreaks, there is a lack of data on integrating viral genome sequences and molecular studies to provide insight into therapeutic options. This is the first study integrating whole genome sequencing of the recent circulating serotype of DENV in 2024 in Saudi Arabia and analyzing the isolate in terms of epidemiology, genomic, mutagenesis, and ligand binding prediction. The most prevalent serotype of DENV in Jeddah city during the study period was DENV-2. Knowing the prevalence of serotypes would have future implications for the design of therapeutics, including the development of vaccines or small molecules that bind to crucial targets in the virus.

Previous studies conducted from 1994–2017 in Jeddah and Makkah showed DENV-2 as a predominant serotype [12,48–50]. Moreover, DENV-2 is also the most common serotype found in the southwestern region of Saudi Arabia (Jazan), as previously reported [29] who, similarly to our study, found no cases of DENV-4. This finding is consistent with recent work by Bello and colleagues (2025), as DENV-2 was the predominant serotype among samples collected from the western and southern regions of Saudi Arabia between 2021 and 2023 [27]. This is of great interest, since one of the largest annual mass-gathering events (Umrah and Hajj) occurs in Saudi Arabia and DENV-2 remains a predominant circulating serotype in the region. The results of this study align with published articles in terms of confirming DENV-2 as the predominant serotype and indicate the necessity for future management to be effective against this serotype. However, our findings are limited by the area in Saudi Arabia and the small sample size. Our study found no cases of DENV-4; however, previous systematic reviews and meta-analyses that include published articles of studies conducted in Saudi Arabia have reported infected subjects (2.38% of the total) with dengue serotype DENV-4 [51]. Continuous analysis of a larger sample size of dengue cases in the region will enable real-time tracking of new DENV genomes as they emerge and tracking any shifts in the prevalent serotypes at the regional level.

The current study’s results indicate that the DENV-2-Jeddah-2024 isolate is in close clustering with strains isolated from Pakistan, China, and Singapore. However, our analysis considered a single genome of one isolate, and the finding cannot be extrapolated to all regional isolates. DENV may have been imported into Saudi Arabia from hyperendemic regions, as millions of travelers and pilgrims visit the country, particularly the Makkah region where Jeddah city is located. In addition, the geographic location of the country, at the meeting point of Africa, Asia, and Europe, could offer an excellent environment for DENV vector *Aedes* mosquitoes [52]. The results of this study support previous findings that the serotype DENV-2 identified in Saudi Arabia was isolated before 2015 and is in close proximity to the strain isolated from Pakistan [20,50]. Furthermore, the recently identified DENV-2 isolate from Jazan in Saudi Arabia showed similarities to DENV-2 sequences reported in Singapore and China, in addition to Malaysia and Korea [28]. The highest proportion of the resident population in Saudi Arabia originate from other Asian countries that are endemic dengue areas [53]. Nevertheless, the highly similar sequences to our isolates indicate that particular nations have a shared ancestry with the currently available references but do not necessarily reflect the location of lineage emergence or definitive geographic origin, given uneven global DENV sequencing and reporting [54].

Generally, females in our study showed a lower risk of dengue incidence than males. Researchers who studied the incidence of dengue in Makkah from 2017 to 2019, reported a consistently higher incidence rate of confirmed dengue cases in males than in females [55]. This is also was reported in the Jazan region in 2020, with a lower incidence of DENV in females compared to males across all DENV serotypes [29]. Religious clothes and the cultural norms of female clothing in the Saudi Arabia region might be one of the reasons for this difference, since clothes can act as a physical barrier to mosquito bites. Moreover, the small sample size of dengue cases in could restrict the generalization of the dominance of males over females as the most infected segment of the population. Research from other countries found no gender-based differences, such as a study conducted in Guangzhou, China, on ~40,000 dengue cases, which found no difference in the risk of dengue infection between males and females [56].

The demographic data of cases reported in the present study indicate that dengue cases mostly occur in young patients (aged 18–39 years). Previous studies in the same region of Saudi Arabia showed that people in the 25–44 years age group were most affected by dengue infection [55,57–60]. This was also reported in different regions of Jazan, where 47.4% of reported DENV was in the age group of 21–40 years [29,61]. However, these findings differ from those of previous studies conducted in other countries, which have suggested that populations under 18 years of age are predominantly affected [34]. The infection risk in Asian countries could increase with age, suggesting that older individuals may be more susceptible to infection or more likely to be exposed to the virus. The relation between age and infection and the differences between regions may relate to many factors, including differences in living or working conditions and immunity acquired among younger people due to previous outbreaks.

In terms of the region of origin, we observed some differences, as Middle Eastern individuals appear to have a higher risk rate for dengue cases compared to other regions. Of the 192 dengue cases included in our study, almost half of the individuals were of Middle Eastern origin (47.4%), followed by participants from South Asia (41.1%), Africa (3.1%), Southeast Asia (1.0%), and South America (0.5%). This observation might be aligned with a significant difference found between Saudi females, who had a higher risk rate for dengue cases, and non-Saudi females [55]. However, another study showed that the majority of dengue fever cases, 66% in 2009 and 77% in 2010, are foreign nationals [62]. This could be attributed to living in subpar neighborhoods due to low economic status, environments that encourage mosquito breeding and increase the risk of developing dengue fever [62,63].

A significant relationship between the number of confirmed dengue cases and the month of Hajj could not be determined in this study. Hajj season occurs annually in the last month (the twelfth month of the Islamic lunar Hijri calendar). This time corresponds to different periods of the Gregorian calendar each year due to the lunar cycle. In 2024, when the sample was collected, the Hajj season was in June, and a total of 23 cases were reported. This number of cases was higher than in most of the other months in the year. However, the result is not significant, and there is a need for a higher sample size. Overall, due to a lack of data and a limited number of studies conducted that cover all regions of the country [51], our results of incidence and demographic characteristics should be interpreted with caution.

Recent literature integrates mutation analysis and clinical data with *in silico* protein modeling and docking studies to observe the association of the role played by mutation with the role of dengue protein [34–36]. NS1-positive samples from dengue cases in Pakistan and India were subjected to serotyping to determine the predominant serotype and characterize the dengue protein for the further exploration of antiviral targets and the identification of potential inhibitors of DENV. Through computational modeling of the envelope protein structure, potential antivirals such as the ligands RGBLD1, RGBLD2, RGBLD3, and RGBLD4, as well as Arbidol and Quercetin, have been proposed to inhibit the early stages of viral infection [34–36]. The epidemiological data in this study were used to perform molecular analysis, as the mutations identified in the locally circulating dengue isolates were used in a docking study. The genomic and mutation analysis were based on a single sample isolate. The *in silico* approach was designed to assess the potential structural and ligand-binding implications of mutations discovered epidemiologically versus the wild-type strain, although these were based on preliminary docking results. The prM protein was chosen as a target model because of availability of the genome as closely related strain to the isolate and its relatively reduced conservation compared to other dengue proteins.

Two mutant residues, D29N and K52N, in isolated prM protein were found in this study. Recent literature has noticed these mutations in prM since 2017 [64]. Previous research has carried out alanine scanning for 26 residues before investigating the antibody-prM contact residues [37]. The residue D29 was not included in the antibody binding, and K52 was not involved in the analysis in the study. Although D29 lies at the antibody binding site, it is not one of the interacting residues with antibody prM13 [37]. Another study performed an epitope-mapping experiment to identify residues crucial for prM-antibody binding [65]. The results showed that mutant K52 did not reduce antibody binding but showed slightly stronger binding than the wild type toward most tested antibodies. In this study, the prM13 antibody showed a similar docking score to the wild type (−22.19) compared to the mutant structure (-22.11) in terms of docking ICM score. Five chemical ligands showed predicted binding to both mutant and wild-type strains that can be tested *in vivo and in vitro* for assessment of efficacy. Nevertheless, these data must be interpreted with caution because of the preliminary nature of the computational work, and they need further validation, molecular dynamics simulation, and *in vitro* and *in vivo* studies.

The present study identified several chemical ligands capable of binding to both the wild-type and mutant forms of the dengue prM protein based on *in silico* predictions. The use of ICM scoring, which combines a GBSA/MM-type energy function with directional hydrogen-bonding terms, enabled robust ranking of hits and the selection of candidates for drug design. The results of this work indicate a general decrease in affinity toward the isolated prM structure. The mutation of the new isolated strain may have caused an alteration in the side-chain orientation and pocket volume, affecting ligand accommodation; however, this is a preliminary docking result, and the functional significance requires further investigation. The study identified new chemical scaffolds (piperidine, azoles, and sulfonamides) targeting reference and mutated prM structures for further investigation. These results may not provide direct therapeutic solutions but improve our knowledge and offer a preliminary molecular insight to aid in the design of targeted antiviral treatments, which may influence future outbreak control. The initial hits presented in this study could guide focused expansion into analogs with the optimization of rich regions of chemical space, which may further improve binding affinities through structure–activity relationship (SAR) studies. Virtual screening of large public databases, such as ZINC, would increase the probability of finding high-affinity hits and diverse chemical scaffolds, as our results are interpreted in the context of the relatively modest set size. Future work might also use recent advances in ultra-large docking workflows, including cloud computing and AI-guided filtering, for the selection of potential candidates. Finally, experimental validation of computational hits remains a crucial next step for the optimization of the identified candidates to evaluate their experimental efficacy as anti-dengue drugs.

In summary, our investigation revealed that during the study period, dengue infection mostly occurred in young non-Saudi males of Middle Eastern origin. Most of the reported cases (95.3%) were caused by DENV serotype-2 (DENV-2). The high sequence identity of the most prevalent serotype (DENV-2) was particularly notable when compared against strains originating from Pakistan (99.68%), Singapore (99.36%), and China (99.37%). This research predicated the 3D structure of the prM as an isolated sequence using AlphaFold2, and the structure was compared with a reference structure (RMSD ranged from 0.63 to 0.92 Å). The research highlighted ligands which are a series of nitrogen-rich scaffolds (piperidine, azoles, and sulfonamides) with diverse substituents as potential binding hits to both receptor structures (wild-type; 8FE4) and mutant (DENV-2-Jeddah-2024). *In silico* mutagenesis followed by docking analysis revealed that the D29N and K52N mutations in the new isolate resulted in predicted lower affinities between identified chemical ligands and the target. Such insights could have implications for designing new anti-dengue drugs in the future and offer insights for possible therapeutic interventions.

## 5. Study limitations

This study was limited to using retrospective data for epidemiological analysis. In addition, the low number of samples and regional isolates may introduce bias when generalizing the results. For the genome analysis, serum samples from DENV-infected patients from a single time were included in this investigation. The results would be strengthened by studies conducted with larger sample sizes and over a period of several years. Our main focus was on analyzing the variations in the DENV-2 virus’s genome, since this was the most common serotype encountered in this study and in several recent studies. Future research could expand the scope by incorporating epidemiological and genomic data and an integrative analysis of mutations across the genomes of multiple DENV serotypes. Furthermore, a single representative isolate was used for the mutation analysis rather than the complete case cohort, which could be addressed in future work. Although *in silico* mutagenesis and computational docking offer useful information on ligand binding with the DENV-2 prM protein, they have some limitations. The complexity of biological settings, including protein flexibility, cellular conditions, and pharmacokinetics, is not taken into consideration by docking studies, which are predictive. The *in silico* analysis and experimental studies on all DENV serotypes, using all drug targets in the virus, could be further explored for therapeutic management. To verify the antiviral properties of the identified ligands and their effectiveness against DENV-2, experimental validation—such as *in vitro* or *in vivo* studies—would be required. However, despite these limitations, the integration of epidemiology and molecular analysis would provide useful information in a “real world” setting and provide an important implication in the context of therapeutic management.

## Supporting information

S1 Table Dengue cases analyzed in the study. (XLSX)

S2 Table Closely similar isolates to our isolated sequence DENV-2-Jeddah-2024 resulting from the BLAST search. (CSV)

S3 File Sequence comparison of QZZ92461 with available sequences using BLAST, highlighting the conservation of residue 137. (ZIP)

S4 Fig Interaction patterns of docked compounds with both prM variants (Left; DENV-JED-2024, right hand; wild type), highlighting key binding residues and types of interactions or contacts. (PNG)

## References

[1] Messina JP, Brady OJ, Golding N, Kraemer MUG, Wint GRW, Ray SE, et al. The current and future global distribution and population at risk of dengue. Nat Microbiol. 2019;4(9):1508–15. doi: 10.1038/s41564-019-0476-8 31182801 PMC6784886

[2] Linard C, Tatem AJ, Gilbert M. Modelling spatial patterns of urban growth in Africa. Appl Geogr. 2013;44:23–32. doi: 10.1016/j.apgeog.2013.07.009 25152552 PMC4139116

[3] Kraemer MUG, Reiner RC Jr, Brady OJ, Messina JP, Gilbert M, Pigott DM, et al. Past and future spread of the arbovirus vectors Aedes aegypti and Aedes albopictus. Nat Microbiol. 2019;4(5):854–63. doi: 10.1038/s41564-019-0376-y 30833735 PMC6522366

[4] Restrepo AC, Baker P, Clements ACA. National spatial and temporal patterns of notified dengue cases, Colombia 2007-2010. Trop Med Int Health. 2014;19(7):863–71. doi: 10.1111/tmi.12325 24862214

[5] Fakeeh M, Zaki A. Dengue in Jeddah, Saudi Arabia, 1994-2002. Dengue Bulletin. 2003;27:13–8.

[6] Sahak MN. Dengue fever as an emerging disease in Afghanistan: Epidemiology of the first reported cases. Int J Infect Dis. 2020;99:23–7. doi: 10.1016/j.ijid.2020.07.033 32738489

[7] Jamal Z, Humayun F, Haider SA, Hakim R, Hill V, Saeed SY, et al. Genomic characterization and serotype distribution of dengue virus circulating in Pakistan during 2024. Infection, Genetics and Evolution. 2025;105815. 10.1016/j.meegid.2025.105815 40876701

[8] Al Balushi L, Al Kalbani M, Al Manji A, Amin M, Al Balushi Z, Al Barwani N, et al. A second local dengue fever outbreak: A field experience from Muscat Governorate in Oman, 2022. IJID Reg. 2023;7:237–41. doi: 10.1016/j.ijregi.2023.03.015 37187798 PMC10176167

[9] Musa AO, Awadap MIM, Faki MTM, Ibrahim HA, Adam A, Siddig EE, et al. Simultaneous circulation of all four dengue virus serotypes during a single outbreak in Kassala, Eastern Sudan 2023. BMC Infect Dis. 2025;25(1):1556. doi: 10.1186/s12879-025-12013-y 41225380 PMC12613636

[10] El Hadad S, Alhebshi A, Al Amri H. Molecular Characterization of Dengue E/NS1 Junction Genotype 2 Isolated From Saudi Patients, Jeddah Province. Pak J Biol Sci. 2018;21(1):38–50. doi: 10.3923/pjbs.2018.38.50 30187718

[11] Al-Raddadi R, Alwafi O, Shabouni O, Akbar N, Alkhalawi M, Ibrahim A, et al. Seroprevalence of dengue fever and the associated sociodemographic, clinical, and environmental factors in Makkah, Madinah, Jeddah, and Jizan, Kingdom of Saudi Arabia. Acta Trop. 2019;189:54–64. doi: 10.1016/j.actatropica.2018.09.009 30244133

[12] Abualamah WA, Banni HS, Almasmoum HA, Allohibi YA, Samarin HM, Bafail MA. Determining Risk Factors for Dengue Fever Severity in Jeddah City, a Case-Control Study (2017). Pol J Microbiol. 2020;69(3):331–7. doi: 10.33073/pjm-2020-036 33574862 PMC7810113

[13] Jamjoom GA, Azhar EI, Kao MA, Radadi RM. Seroepidemiology of asymptomatic dengue virus infection in Jeddah, Saudi Arabia. Virology: Research and Treatment. 2016;7:VRT. 10.4137/VRT.S34187 PMC4758801 26917954

[14] Hashem AM, Abujamel T, Alhabbab R, Almazroui M, Azhar EI. Dengue infection in patients with febrile illness and its relationship to climate factors: A case study in the city of Jeddah, Saudi Arabia, for the period 2010-2014. Acta Trop. 2018;181:105–11. doi: 10.1016/j.actatropica.2018.02.014 29452109

[15] Alhaeli A, Bahkali S, Ali A, Househ MS, El-Metwally AA. The epidemiology of Dengue fever in Saudi Arabia: A systematic review. J Infect Public Health. 2016;9(2):117–24. doi: 10.1016/j.jiph.2015.05.006 26106040

[16] Alkhaldy I, Barnett R. Explaining Neighbourhood Variations in the Incidence of Dengue Fever in Jeddah City, Saudi Arabia. Int J Environ Res Public Health. 2021;18(24):13220. doi: 10.3390/ijerph182413220 34948849 PMC8706944

[17] Hegazi MA, Bakarman MA, Alahmadi TS, Butt NS, Alqahtani AM, Aljedaani BS, et al. Risk Factors and Predictors of Severe Dengue in Saudi Population in Jeddah, Western Saudi Arabia: A Retrospective Study. Am J Trop Med Hyg. 2020;102(3):613–21. doi: 10.4269/ajtmh.19-0650 31933467 PMC7056408

[18] Al-Nefaie H, Alsultan A, Abusaris R. Temporal and spatial patterns of dengue geographical distribution in Jeddah, Saudi Arabia. J Infect Public Health. 2022;15(9):1025–35. doi: 10.1016/j.jiph.2022.08.003 36007387

[19] Alyahya HS. Prevalence of dengue fever in Saudi Arabia: Jeddah as a case study. Entomological Research. 2023;53(12):539–53. doi: 10.1111/1748-5967.12685

[20] Azhar EI, Hashem AM, El-Kafrawy SA, Abol-Ela S, Abd-Alla AMM, Sohrab SS, et al. Complete genome sequencing and phylogenetic analysis of dengue type 1 virus isolated from Jeddah, Saudi Arabia. Virol J. 2015;12:1. doi: 10.1186/s12985-014-0235-7 25591713 PMC4310205

[21] Hashem AM, Sohrab SS, El-Kafrawy SA, Abd-Alla AMM, El-Ela SA, Abujamel TS, et al. Diversity of dengue virus-3 genotype III in Jeddah, Saudi Arabia. Acta Trop. 2018;183:114–8. doi: 10.1016/j.actatropica.2018.04.002 29621534

[22] Hashem AM, Sohrab SS, El-Kafrawy SA, El-Ela SA, Abd-Alla AMM, Farraj SA, et al. First complete genome sequence of circulating dengue virus serotype 3 in Jeddah, Saudi Arabia. New Microbes New Infect. 2017;21:9–11. doi: 10.1016/j.nmni.2017.09.005 29158909 PMC5678886

[23] GASTAT. GASTAT General Authority for Statistic announces total number of Umrah performers and pilgrims for 1443/2022. https://www.stats.gov.sa/en/w/gastat-announces-total-number-of-umrah-performers-and-pilgrims-for-1443-/2022 2026 March 26.

[24] GASTAT. General Authority for Statistic Hajj Report 1444/2023. 2023. https://www.stats.gov.sa/documents/20117/1400941/Hajj±Statistics±2023±EN.pdf/96d002f0-2fa4-4c67-b334-ef19a4604b63?t=1734112849496

[25] Harapan H, Michie A, Mudatsir M, Sasmono RT, Imrie A. Epidemiology of dengue hemorrhagic fever in Indonesia: analysis of five decades data from the National Disease Surveillance. BMC Res Notes. 2019;12(1):350. doi: 10.1186/s13104-019-4379-9 31221186 PMC6587249

[26] Singh N, Singh AK, Kumar A. Dengue outbreak update in India: 2022. Indian Journal of Public Health. 2023;67(1):181–3. 37039229 10.4103/ijph.ijph_1517_22

[27] Bello MB, BuAli Z, Trovao NS, Aljedani SS, Algaissi A, Shrwani KJ, et al. Molecular evolutionary insights into the repeated introductions and cryptic transmission of dengue virus in Saudi Arabia. J Infect. 2025;91(3):106608. doi: 10.1016/j.jinf.2025.106608 40907669

[28] Dafalla O, Abdulhaq AA, Almutairi H, Noureldin E, Ghzwani J, Mashi O, et al. The emergence of an imported variant of dengue virus serotype 2 in the Jazan region, southwestern Saudi Arabia. Trop Dis Travel Med Vaccines. 2023;9(1):5. doi: 10.1186/s40794-023-00188-8 36922890 PMC10018863

[29] Ommer D, Abdulaziz H, Elsiddig N, Siddig A, Yehya H, Tareq K, et al. Distribution of Dengue Virus Serotypes in Jazan Region, Southwest Saudi Arabia. Ann Public Health Reports. 2021;5(2). doi: 10.36959/856/520

[30] Manack A, C C, Kaplowitz H. Role of Epidemiological Data Within the Drug Development Lifecycle: A Chronic Migraine Case Study. Epidemiology - Current Perspectives on Research and Practice. InTech. 2012. doi: 10.5772/32343

[31] Peng L, Shen L, Xu J, Tian X, Liu F, Wang J, et al. Prioritizing antiviral drugs against SARS-CoV-2 by integrating viral complete genome sequences and drug chemical structures. Sci Rep. 2021;11(1):6248. doi: 10.1038/s41598-021-83737-5 33737523 PMC7973547

[32] Moeini S, Mohebbi A, Farahmand B, Mehrbod P, Fotouhi F. Phylogenetic analysis and docking study of neuraminidase gene of influenza A/H1N1 viruses circulating in Iran from 2010 to 2019. Virus Res. 2023;334:199182. doi: 10.1016/j.virusres.2023.199182 37490957 PMC10407273

[33] Brcko IC, de Souza VC, Ribeiro G, Lima ARJ, Martins AJ, Barros CRDS, et al. Comprehensive molecular epidemiology of influenza viruses in Brazil: insights from a nationwide analysis. Virus Evol. 2024;11(1):veae102. doi: 10.1093/ve/veae102 39802823 PMC11711486

[34] Racherla RG, Katari SK, Mohan A, Amineni U, Badur M, Chaudhury A, et al. Molecular Characterization and Identification of Potential Inhibitors for “E” Protein of Dengue Virus. Viruses. 2022;14(5):940. doi: 10.3390/v14050940 35632682 PMC9143040

[35] Ali H, Saleem I, Ahmed MS, Amraiz D, Shahid I, Al-Shahari EA, et al. Dominance of dengue virus serotype-2 in Pakistan (2023–2024): Molecular characterization of the envelope gene and exploration of antiviral targets. Virus Research. 2024;350:199497. 39557198 10.1016/j.virusres.2024.199497 PMC11625376

[36] Ravi V, Imran M, Khare K, Mishra P, Mohite R, Kanika, et al. Clinico-genomic study reveals association of dengue virus genome high frequency mutations with dengue disease severity. Sci Rep. 2025;15(1):18724. doi: 10.1038/s41598-025-00462-z 40436903 PMC12120001

[37] Dowd KA, Sirohi D, Speer SD, VanBlargan LA, Chen RE, Mukherjee S, et al. prM-reactive antibodies reveal a role for partially mature virions in dengue virus pathogenesis. Proc Natl Acad Sci U S A. 2023;120(3):e2218899120. doi: 10.1073/pnas.2218899120 36638211 PMC9933121

[38] Altschul SF, Gish W, Miller W, Myers EW, Lipman DJ. Basic local alignment search tool. J Mol Biol. 1990;215(3):403–10. doi: 10.1016/S0022-2836(05)80360-2 2231712

[39] Madeira F, Madhusoodanan N, Lee J, Eusebi A, Niewielska A, Tivey ARN, et al. The EMBL-EBI Job Dispatcher sequence analysis tools framework in 2024. Nucleic Acids Res. 2024;52(W1):W521–5. doi: 10.1093/nar/gkae241 38597606 PMC11223882

[40] Lemoine F, Correia D, Lefort V, Doppelt-Azeroual O, Mareuil F, Cohen-Boulakia S, et al. NGPhylogeny.fr: new generation phylogenetic services for non-specialists. Nucleic Acids Res. 2019;47(W1):W260–5. doi: 10.1093/nar/gkz303 31028399 PMC6602494

[41] Mirdita M, Schütze K, Moriwaki Y, Heo L, Ovchinnikov S, Steinegger M. ColabFold: making protein folding accessible to all. Nat Methods. 2022;19(6):679–82. doi: 10.1038/s41592-022-01488-1 35637307 PMC9184281

[42] Jumper J, Evans R, Pritzel A, Green T, Figurnov M, Ronneberger O, et al. Highly accurate protein structure prediction with AlphaFold. Nature. 2021;596(7873):583–9. doi: 10.1038/s41586-021-03819-2 34265844 PMC8371605

[43] Neurosnap. Neurosnap: An online platform for computational biology and chemistry. https://neurosnap.ai/ 2022.

[44] Abagyan R, Totrov M, Kuznetsov D. ICM—A new method for protein modeling and design: Applications to docking and structure prediction from the distorted native conformation. J Comput Chem. 1994;15(5):488–506. doi: 10.1002/jcc.540150503

[45] Molsoft L. Protein-protein docking tutorial using FFT method. https://www.molsoft.com/icmpro/protein-protein-docking-tutorial.html#gsc.tab=0 2026.

[46] Cox OB, Krojer T, Collins P, Monteiro O, Talon R, Bradley A, et al. A poised fragment library enables rapid synthetic expansion yielding the first reported inhibitors of PHIP(2), an atypical bromodomain. Chem Sci. 2016;7(3):2322–30. doi: 10.1039/c5sc03115j 29910922 PMC5977933

[47] Molsoft L. ICM User’s Guide Small Molecule Docking. https://www.molsoft.com/gui/start-dock.html#vls-score 2025.

[48] Fakeeh M, Zaki A. Virologic and serologic surveillance for dengue fever in Jeddah, Saudi Arabia, 1994-1999. The American Journal of Tropical Medicine and Hygiene. 2001;65(6):764–7. 11791972 10.4269/ajtmh.2001.65.764

[49] Ashshi AM. The prevalence of dengue virus serotypes in asymptomatic blood donors reveals the emergence of serotype 4 in Saudi Arabia. Virol J. 2017;14(1):107. doi: 10.1186/s12985-017-0768-7 28599678 PMC5466713

[50] Al‐Saeed MS, El‐Kafrawy SA, Farraj SA, Al‐Subhi TL, Othman NA, Alsultan A, et al. Phylogenetic characterization of Circulating dengue and Alkhumra hemorrhagic fever viruses in Western Saudi Arabia and lack of evidence of Zika virus in the region: A retrospective study, 2010‐2015. Journal of medical virology. 2017;89(8):1339–46. 28198548 10.1002/jmv.24785 PMC7167144

[51] Rabaan AA, Alshengeti A, Alrasheed HA, Al-Subaie MF, Aljohani MH, Almutawif YA, et al. Dengue virus infection in Saudi Arabia from 2003 to 2023: a systematic review and meta-analysis. Pathog Glob Health. 2024;118(7–8):549–58. doi: 10.1080/20477724.2024.2425493 39508063 PMC11892044

[52] Alahmed AM, Munawar K, Khalil SMS, Harbach RE. Assessment and an updated list of the mosquitoes of Saudi Arabia. Parasit Vectors. 2019;12(1):356. doi: 10.1186/s13071-019-3579-4 31324201 PMC6642568

[53] Altassan KK, Morin C, Shocket MS, Ebi K, Hess J. Dengue fever in Saudi Arabia: A review of environmental and population factors impacting emergence and spread. Travel Med Infect Dis. 2019;30:46–53. doi: 10.1016/j.tmaid.2019.04.006 30978417

[54] WHO. Dengue - Global situation 2024. https://www.who.int/emergencies/disease-outbreak-news/item/2024-DON518 2026 February 20.

[55] Melebari S, Bakri R, Hafiz A, Qabbani F, Khogeer A, Alharthi I, et al. The epidemiology and incidence of dengue in Makkah, Saudi Arabia, during 2017-2019. Saudi Med J. 2021;42(11):1173–9. doi: 10.15537/smj.2021.42.11.20210124 34732548 PMC9149740

[56] Jiang L, Liu Y, Su W, Liu W, Dong Z, Long Y, et al. Epidemiological and genomic analysis of dengue cases in Guangzhou, China, from 2010 to 2019. Sci Rep. 2023;13(1):2161. doi: 10.1038/s41598-023-28453-y 36750601 PMC9905598

[57] Alzahrani AG, Al Mazroa MA, Alrabeah AM, Ibrahim AM, Mokdad AH, Memish ZA. Geographical distribution and spatio-temporal patterns of dengue cases in Jeddah Governorate from 2006-2008. Trans R Soc Trop Med Hyg. 2013;107(1):23–9. doi: 10.1093/trstmh/trs011 23222946

[58] Khan NA, Azhar EI, El-Fiky S, Madani HH, Abuljadial MA, Ashshi AM, et al. Clinical profile and outcome of hospitalized patients during first outbreak of dengue in Makkah, Saudi Arabia. Acta Trop. 2008;105(1):39–44. doi: 10.1016/j.actatropica.2007.09.005 17983609

[59] Ayyub M, Khazindar AM, Lubbad EH, Barlas S, Alfi AY, Al-Ukayli S. Characteristics of dengue fever in a large public hospital, Jeddah, Saudi Arabia. J Ayub Med Coll Abbottabad. 2006;18(2):9–13. 16977805

[60] Kholedi AAN, Balubaid O, Milaat W, Kabbash IA, Ibrahim A. Factors associated with the spread of dengue fever in Jeddah Governorate, Saudi Arabia. East Mediterr Health J. 2012;18(1):15–23. doi: 10.26719/2012.18.1.15 22360006

[61] Al-Azraqi TA, El Mekki AA, Mahfouz AA. Seroprevalence of dengue virus infection in Aseer and Jizan regions, Southwestern Saudi Arabia. Transactions of the Royal Society of Tropical Medicine and Hygiene. 2013;107(6):368–71. 23474472 10.1093/trstmh/trt022

[62] Khormi HM, Kumar L. Assessing the risk for dengue fever based on socioeconomic and environmental variables in a geographical information system environment. Geospat Health. 2012;6(2):171–6. doi: 10.4081/gh.2012.135 22639119

[63] Khormi HM, Kumar L, Elzahrany RA. Modeling spatio-temporal risk changes in the incidence of Dengue fever in Saudi Arabia: a geographical information system case study. Geospat Health. 2011;6(1):77–84. doi: 10.4081/gh.2011.159 22109865

[64] Alam S, Tony SR, Khair S, Punno HT, Begum MN, Rahman M. Epitope dynamics and antigenic shifts in dengue virus serotype 2 cosmopolitan genotype with structural changes in Bangladesh from 2017 to 2023. Scientific Reports. 2026. 10.1038/s41598-026-38446-2 PMC13057000 41764225

[65] Keelapang P, Supasa P, Sriburi R, Puttikhunt C, Cardosa J, Kasinrerk W, et al. A group of infection-enhancing and focus size-reducing monoclonal antibodies recognized an “a and c” strands epitope in the pr domain of Dengue Virus prM. Virus Res. 2023;323:199015. doi: 10.1016/j.virusres.2022.199015 36455752 PMC9742851

[66] Dafalla OMaE. Polyprotein, partial [dengue virus type 2]. 2021.

