# Supplementary figures and images for: Epidemiological and genomic analysis of the dengue virus isolate from Jeddah, Saudi Arabia: Implications for future therapeutic development
Source: PLoS One. 2026 Jul 16;21(7):e0351649. doi: 10.1371/journal.pone.0351649 (PMC13375040; doi:10.1371/journal.pone.0351649)

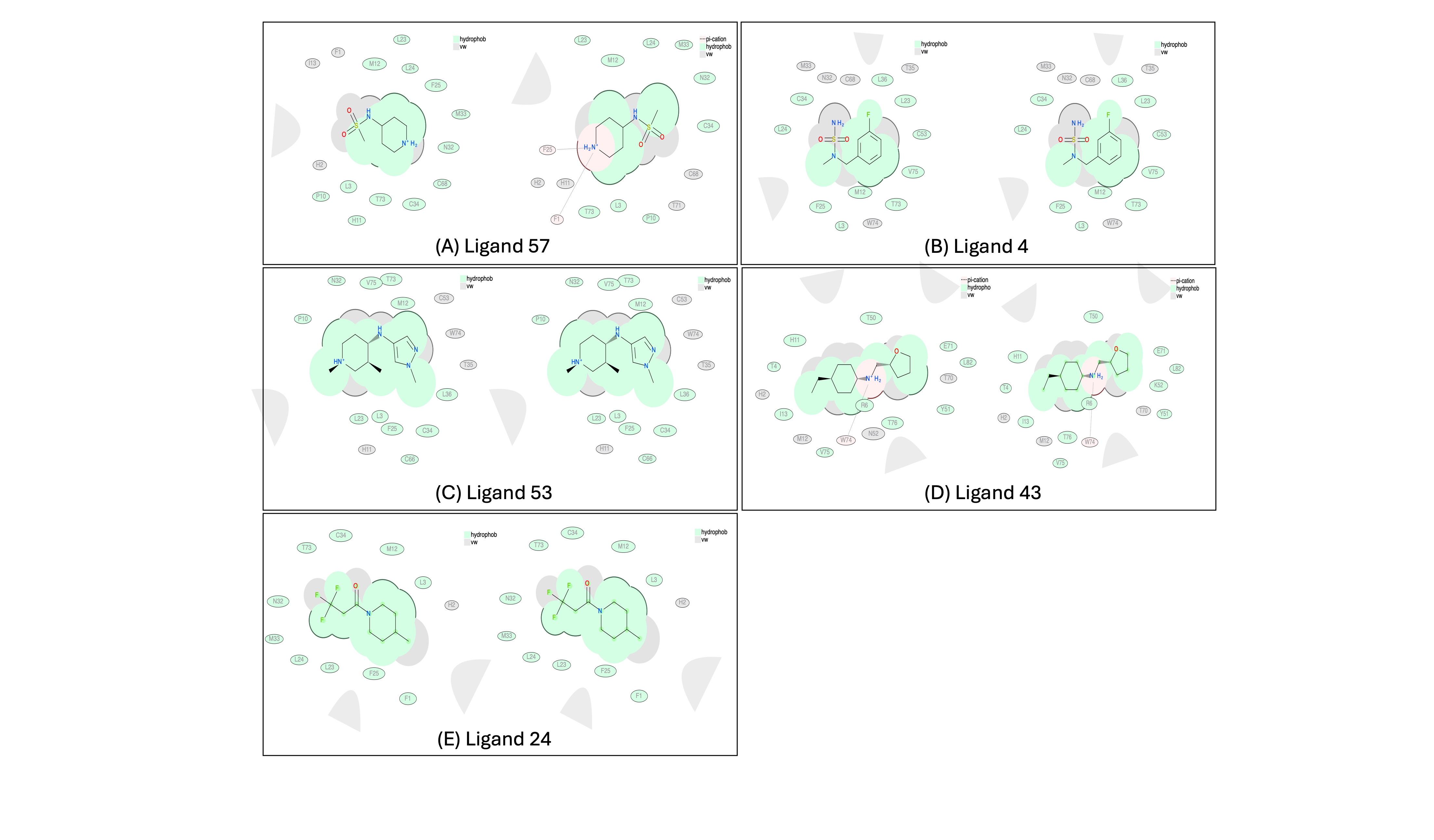

Supplement: S4 Fig — (PNG) [file pone.0351649.s004.png]
